# Radioprotective effect of a combination of melatonin and metformin on mice spermatogenesis: A histological study

**DOI:** 10.18502/ijrm.v18i12.8029

**Published:** 2020-12-21

**Authors:** Elham Tajabadi, Abdolreza Javadi, Nasim Ahmadi Azar, Masoud Najafi, Alireza Shirazi, Dheyauldeen Shabeeb, Ahmed Eleojo Musa

**Affiliations:** ^1^Department of Medical Radiation Engineering, Science and Research Branch, Islamic Azad University, Tehran, Iran.; ^2^Department of Pathology, Imam Hossein Hospital, Shahid Beheshti University of Medical Sciences, Tehran, Iran.; ^3^Medical Technology Research Center, Institute of Health Technology, Kermanshah University of Medical Sciences, Kermanshah, Iran.; ^4^Radiology and Nuclear Medicine Department, School of Paramedical Sciences, Kermanshah University of Medical Sciences, Kermanshah, Iran.; ^5^Department of Medical Physics and Biomedical Engineering, Faculty of Medicine, Tehran University of Medical Sciences, Tehran, Iran.; ^6^Radiation Oncology Department, Cancer Institute, Tehran University of Medical Sciences, Tehran, Iran.; ^7^Department of Physiology, College of Medicine, University of Misan, Misan, Iraq.; ^8^Research Center for Molecular and Cellular Imaging, Tehran University of Medical Sciences (International Campus), Tehran, Iran.

**Keywords:** Radiation, Testis, Leydig cells, Melatonin, Metformin, Spermatogenesis.

## Abstract

**Background:**

The spermatogenesis system includes highly radiosensitive cells. Hence, this system is a potential target for toxic effects of ionizing radiation during radiotherapy of abdomen and pelvis cancers, as well as after accidental radiation events. Accordingly, metformin and melatonin are two important radioprotectors that have shown an ability to prevent cell death through neutralization of free radicals and stimulating DNA damage responses.

**Objective:**

To evaluate the radioprotective effects of melatonin and metformin on mice spermatogenesis when administered alone or as a combination.

**Materials and Methods:**

In this histological Study, 40 (6-8 wk, 30 gr) NMRI mice were divided into 8 groups (n = 5/each) as control, metformin, melatonin, melatonin + metformin, radiation, radiation + melatonin, radiation + metformin, and radiation + melatonin + metformin. 37 days after the irradiation, the testicular tissues were collected for histological evaluation.

**Results:**

Single administration of melatonin could ameliorate effectively radiation toxicity in mice testis. Metformin showed radioprotective effects on some parameters such as the numbers of spermatogonia and mature sperms. Interestingly, the melatonin and metformin combination reversed the reduced number of sperms rather than single drug administration.

**Conclusion:**

The combination of melatonin with metformin can protect mice spermatogenesis against ionizing radiation more effectively compared to the single forms of these drugs.

## 1. Introduction

In today's world, ionizing radiation plays a vital role in human life. Medical applications of ionizing radiation including radiotherapy and diagnostic radiology are major benefits of radiation to man. It has also been utilized for agricultural and industrial purposes (1). Besides the useful applications of radiation, its exposure could lead to detrimental consequences to the human body. The major aim of radiotherapy is tumor eradication. However, it has been associated with toxicity to adjacent organs (2). In addition to the useful applications of ionizing radiation, there has also been the threat of its use for terror aims, as well as accidental radiation events such as explosions from nuclear stations (3). Bone marrow hematopoietic cells, gastrointestinal and spermatogenesis systems are the most radiosensitive organs. Depletion of stem cells in the bone marrow and gastrointestinal system occurs following exposure to radiation doses higher than 4 Gy, which may lead to death (4). However, spermatogenic arrest may be observed following exposure to lower radiation doses (5). The testis is made up of various cell types with different characteristics. It has been proposed that the spermatogonia (germinal cells within the testis) are the most radiosensitive cells (6). The spermatogonia differentiate to spermatids, which produce spermatocytes and finally mature sperm cells. In addition to spermatogonia, other cells are highly sensitive to radiation with lethal dose 37% (LD 37%) lower than 1 Gy (7). Hence, exposure to sublethal doses of radiation (1-4 Gy), which is common during radiotherapy or a radiation disaster, can lead to potent suppression of spermatogenesis. This issue is very important for patients with pelvic cancers, whereby the testis is within or adjacent to the radiation field (8). One of the most common and easiest strategies for amelioration of toxic damage to spermatogenesis in response to radiation exposure is the use of some agents which reduce radiation-induced DNA damages as well as cell death (9).

In the present study, we examined the possible protective effects of melatonin and metformin against radiation-induced mice spermatogenesis injury.

## 2. Materials and Methods

### Experimental design

In this experimental study, 40 NMRI mice (6-8 wk, 30 gr) were divided into eight groups as follows (n = 5/each); G1 (control) recieved only ketamine and xylazine for anesthesia similar to other groups; G2 (metformin treated) received only 100 mg/kg metformin; G3 (melatonin treated) received only 100 mg/kg melatonin; G4 (melatonin + metformin) received 100 mg/kg metformin + 100 mg/kg melatonin; G5: (radiation only group) were irradiated with 2 Gy gamma rays to whole body without any drug treatment; G6: (radiation and metformin) were irradiated locally to pelvis area with 2 Gy gamma rays and also received 100 mg/kg metformin; G7 (radiation and melatonin were irradiated locally to pelvis area with 2 Gy gamma rays and also received 100 mg/kg melatonin; G8 (radiation and combination of melatonin and metformin) were irradiated locally to pelvis area with 2 Gy gamma rays and also received metformin and melatonin at 100 mg/kg. Next, 37 days after the irradiation, all mice were sacrificed. Their right testes were then removed and fixed in Bouin's solution for histopathological evaluation.

### Drugs and irradiation

Melatonin provided from Merck company (Germany). Melatonin powder dissolved in ethanol and then diluted in distilled water. Final our solution was included 15% ethanol and the concentration of melatonin was 3 mg per each milliliter. Metformin was purchased from Tehran Chiemi Company, Tehran, Iran. As metformin is soluble in water, it dissolved in distilled water at a similar concentration. For preparing the melatonin and metformin combination, melatonin was first dissolved in ethanol, and then diluted in distilled water. Afterward, metformin was added to the melatonin solution. The concentration of both melatonin and metformin was 3 mg/ml. Treatments commenced two days before irradiation at one dose/day. The last treatment was administered 30 min before irradiation. Beside, melatonin and metformin as well as their combination were administered orally at 100 mg/kg. These drug doses were chosen similar to some previous animal studies (10, 11).

Irradiation was done by a cobalt-60 (60°C) gamma rays source. Before irradiation, all mice were anesthetized using ketamine and xylazine of doses 80 and 5 mg/kg, respectively. Next, the mice were positioned supine and irradiated with 60°C gamma rays (1.25 MeV). Irradiation was done with 2 Gy gamma rays at a dose rate of 60 cGy/min as a single dose. The selection of 2 Gy dose of radiation was based on a previous study on murine spermatogenesis system (12).

### Histopathology 

Tissue samples were provided for light microscopy examination according to standard procedures. The testes were then fixed in Bouin's solution, then, provided as small piecesin paraffin wax. These samples sectioned at 3 μm, and then stained with hematoxylin and eosin (a common method for evaluating morphological changes) and PAS special staining (for assessment of basement membrane thickness). Testis tissue slides were evaluated for common morphological changes in spermatogenesis including numbers of sperms and spermatogonia, spermatogenic arrest, damage to seminiferous tubules, interstitial edema, Leydig cell hyperplasia (13-15), and Johnsen scoring (16). The results of histopathological evaluation were reported using the following scores: normal = 0; mild = 1; moderate = 2; and severe = 3.

### Ethical consideration

This study was approved by the Tehran University of Medical Sciences, under ethical code: IR.TUMS.MEDICINE.REC.1396.3787. All mice received enough anesthesiza drugs before scarification to prevent a painful death.

### Statistical analysis

After scoring, results were reported as mean ± standard deviation (mean ± SD). To determine the statistical significance between groups, the SPSS software version 16 (IBM Chicago, USA) was used. Additionally, the Mann-Whitney test was performed to analyze the statistical differences between groups. P-value < 0.05 was considered as statistically significant.

## 3. Results

**Figure 1:**
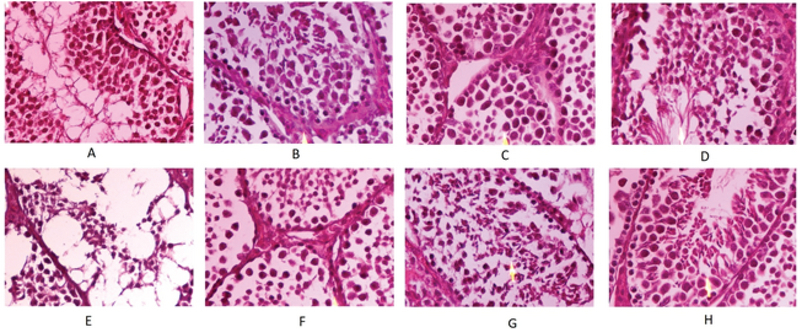
(A-H) Photomicrographs of radioprotective effect of melatonin, metformin, and their combination on mice spermatogenesis. (H and E ×1000) (A) Normal spermatogenesis in the control group, JS* = 9; (B) Spermatogenesis in melatonin group, average JS* = 9-10; (C) Spermatogenesis within normal limit in metformin group, average JS* = 9-10; (D) Spermatogenesis in melatonin + metformin group, JS* = 9; (E) Nearly all lineage of spermatogonial cells were damaged in the radiation only group. Severe tubular atrophy, decreased number of germ cells per tubules (round empty spaces), and few spermatocytes were present, JS* = 4; (F) Moderate decrease in spermatogenesis with only few spermatids in the radiation + melatonin group, JS* = 6; (G) Mild decrease in the number of germ cells within seminiferous tubules in the radiation + metformin group. A few spermatids were present (marked with arrow), JS* = 7; (H) Sub-normal spermatogenesis in the radiation + melatonin + metformin group, some spermatozoa are seen within the lumen (marked with arrow), average JS* = 8. *Johnsen scoring.

Our assesments confirmed that exposure of mice to 2 Gy gamma rays causes mild to severe damages to spermatogenesis system. Irradiation also gave rise to moderate atrophy of seminiferous tubules, interstitial edema, and Leydig cell hyperplasia. Treatment with melatonin or its combination with metformin was able to ameliorate atrophy of seminiferous tubules, while administering metformin alone could not protect them. Neither drug could mitigate interstitial edema, although they could reduce radiation-induced Leydig cell hyperplasia. In addition, treatment with drugs did not show any effect on spermatogenic arrest, while irradiation led to mild thickening of basal lamina. Basal lamina thickening was ameliorated with melatonin as well as its combination with metformin; however, metformin treatment alone could not mitigate it. Irradiation caused severe reduction of spermatogonia numbers and Johnsen score. Treatment with melatonin, metformin, as well as their combination was able to alleviate these reductions. Histological evaluation of epididymis showed a severe vacuolation with no detectable mature sperm. Mice treated with melatonin or metformin had few spermatozoa. Interestingly, mice treated with the combination of melatonin and metformin showed normal epididymis (Figure 1, Table I).

**Table 1:** Results of morphological changes following irradiation with or without melatonin, metformin or their combination in mice testes


	**Control**	**MLT**	**MET**	**MLT + MET**	**RAD**	**RAD + MLT**	**RAD + MET**	**RAD + MLT + MET**
**Atrophy of seminiferous tubules**	0.00 ± 00	0.00 ± 00	0.00 ± 00	0.00 ± 00	2.00 ± 1.0${}^{a}$	0.80 ± 0.83${}^{b}$	1.00 ± 00	0.60 ± 0.54${}^{b,c}$
**Interstitial edema**	0.00 ± 00	0.60 ± 0.54	0.60 ± 0.54	0.80 ± 0.44	2.00 ± 00	1.40 ± 0.89	1.75 ± 0.50	1.00 ± 0.57
**Spermatogenic arrest**	0.00 ± 00	0.00 ± 00	0.00 ± 00	0.00 ± 00	4.00 ± 00${}^{a}$	0.00 ± 00	0.00 ± 00	0.00 ± 00
**Thickening of basal lamina**	0.00 ± 00	0.00 ± 00	0.00 ± 00	0.00 ± 00	1.00 ± 00${}^{a}$	0.44 ± 0.20${}^{b,c}$	1.00 ± 00	0.44 ± 0.20${}^{b,c}$
**Leydig cell hyperplasia **	0.00 ± 00	0.00 ± 00	0.33 ± 0.57	0.40 ± 0.54	2.00 ± 00${}^{a}$	0.00 ± 00${}^{b}$	0.40 ± 0.54${}^{b}$	0.20 ± 0.44${}^{b}$
**Number of spermatogonia**	325 ± 27	337 ± 13	327 ± 11	325 ± 39	74 ± 9${}^{a}$	187 ± 11${}^{b,c}$	154 ± 8${}^{b}$	199 ± 11${}^{b,c}$
**Johnsen scoring**	8.80 ± 0.44	9.00 ± 0.70	8.60 ± 0.54	8.60 ± 0.54	4.00 ± 00${}^{a}$	6.80 ± 1.09${}^{b}$	7.00 ± 1.15${}^{b}$	7.28 ± 0.95${}^{b}$
**Epididymis damage**	Normal	Normal	Normal	Normal	Vacuolation and no sperm	Few spermatozoa	Few spermatozoa	Normal
Data presented as Mean ± SD. Mann-Whitney test, p < 0.05, ${}^{a}$ Significant compared to control group; ${}^{b}$ Significant compared to radiation (RAD) group; ${}^{c}$ Significant compared to radiation + metformin (RAD + MET) group. Comparisons between RAD + MLT and RAD + MLT + MET was not significant, MLT: Melatonin, MET: Metformin, RAD: Radiation								

## 4. Discussion

Ionizing radiation has potent detrimental effects on spermatogenesis system. A low to mild dose of radiation is able to destroy normal spermatogenesis via induction of apoptosis in germ cells and spermatogonia. Even a sublethal dose of ionizing radiation (e.g., 1-4 Gy) can cause temporary or permanent infertility. Hence, exposure to a sublethal dose of radiation such as seen in accidental nuclear or radiological events can cause potent inhibition of the reproductive system (17). Moreover, during pelvic or abdomen radiotherapy, scattered radiation is able to cause massive damage to spermatogenesis. The changes in the level of sex hormones such as follicle-stimulating hormone and testosterone amplifies spermatogenesis damage following radiotherapy (18). Low level of testosterone following low- and high doses of ionizing radiation can disrupt maturation of primary spermatids. Decreased testosterone in association with unusual follicle-stimulating hormone and luteinizing hormone can be a sign of damage to spermatogenesis, which can be observed in patients that have undergone radiotherapy for pelvic cancers (19).

To Infertility isn't only concern, and exposure of the testis to ionizing radiation may increases risk of carcinogenesis in future generations. Several experiments have confirmed some evidences for delayed consequences of radiation in next generation of irradiated cells. These pieces of evidence indicate that radiation induces cancer in future generations of irradiated cells. Furthermore, an increase in the expression of oncogenes has reported in the next generation (20). The highly radiosensitive nature of spermatogenesis system has been confirmed among people who were exposed to sublethal doses of radiation. Møller and colleagues showed that the frequency of aberrant morphology in the sperms of Chernobyl survivors was higher than normal levels. They also showed that this has a direct relationship with serum level of antioxidants, including retinol, α-tocopherol, and carotenoids (21). Another study showed changes in the characteristics of sperm function, increased DNA damage, and hypermethylation among radiation workers (22). Due to the high radiosensitivity of spermatogenesis system, several studies have been conducted to introduce a safe drug or other agents for protection against ionizing radiation. Previous studies have revealed that antioxidants such as ascorbic acid and alpha-tocopherol can protect against ionizing radiation-induced toxic damage to spermatogenesis (12, 23).

Our results showed that whole body exposure to radiation led to damage to seminiferous tubules and epididymis, edema in interstitial, and remarkable damage to spermatogenesis and Leydig cells. Severe damage to spermatogonia and reduction in the numbers of spermatids and sperms are signs of spermatogenic arrest, which was obvious following irradiation. In the present study, we showed that the combination of melatonin and metformin has a potent radioprotection on mice spermatogenesis without a significant cytotoxicity. Melatonin is a pineal hormone, regulated in a circadian manner, however, several studies have suggested other regulatory roles for it. Melatonin, as a drug, is used to improve sleep. Also, the use of melatonin as an adjuvant for other diseases may lead to drowsiness (24). Studies have shown that melatonin is able to neutralize different types of free radicals directly or via activation of antioxidant enzymes such as superoxide dismutase (SOD), glutathione (GSH), glutathione peroxidase (GPx), catalase (CAT), and glutathione reductase (GR) (25). Several bodies of evidence have suggested that melatonin inhibits reduction/oxidation reactions following exposure to radiation, leading to inhibition of chronic oxidative stress in irradiated cells (26). A systematic review by Zetner and colleagues proposed that melatonin can be potentially used for cancer patients undergoing radiotherapy (27). The differential effect of melatonin on normal tissues against some cancer cells also make it a potential adjuvant for both radioprotection of normal tissues and radiosensitization of cancers (25). Administration of melatonin before exposure to ionizing radiation has been shown to reduce apoptosis in rat's testis and attenuates the expression of caspase-3 in primary spermatocytes (28). The administration of melatonin has also been shown to ameliorate damage to Sertoli cells. Furthermore, it induces antioxidant defense and increases proliferation of spermatogenesis, which lead to reduction of toxicity in testis following irradiation or exposure to chemotherapy (29, 30).

Metformin is another agent that has shown promising radioprotective effects. It is an FDA- approved antidiabetic drug which is used by millions of people worldwide. Metformin has shown ability to scavenge free radicals and stimulate antioxidant enzymes, thus reducing apoptosis and genotoxicity, leading to amelioration of cell death (31). In addition, via inhibition of electron transfer chain 1 (ETC1) in the mitochondria, it attenuates redox activity and continuous production of superoxide by mitochondria during oxidative stress conditions (32). Emerging evidence shows that in addition to reducing the risk of carcinogenesis, metformin can also be proposed as an adjuvant in cancer therapy (33). It is important to note that metformin also has some side effects. The most important side effects include hypoglycemia and weakness, however, in some situation it may cause lactic acidosis, pain, or infection (34). A previous study showed that metformin is able to reduce apoptosis and damage to basal lamina following ischemia/reperfusion in rats (35). Results of this study showed that melatonin, when administered alone or in combination with metformin, can ameliorate all evaluated damages except interstitial edema. Metformin also showed an ability to increase the numbers of spermatogonia and mature sperms, as well as alleviate damages to Leydig cells. The effect of the combination of melatonin with metformin was similar to that of melatonin administered alone. However, the combination was more potent in preserving mature sperms compared with melatonin or metformin alone. Moreover, single treatment with melatonin was more potent compared to that of metformin in preserving most of the spermatogenic activity. It seems that via stimulation of DNA damage repair, melatonin and metformin were able to protect the spermatogonia and other testicular cells such as spermatids, spermatocytes, and Leydig cells against massive DNA damage and cell death. Interestingly, results of histological evaluation suggest that the combination of both melatonin and metformin may have a synergic effect in protecting these progenitor cells against the clastogenic effects of ionizing radiation.

## 5. Conclusion

This study showed that the combination of melatonin with metformin can protect against ionizing radiation-induced damage to spermatogenesis more effectively compared to the single forms of these drugs. Moreover, results of this study showed that the radioprotective effect of melatonin is more potent compared to metformin's. It is possible that the combination of these drugs has a synergic effect on the protection of immature spermatogonia or other sperm progenitor cells against genotoxic effects of ionizing radiation, however, this will require further studies.

##  Conflict of Interest

The authors declare that there is no conflict of interest.
